# Structure and vibrational spectroscopy of methanesulfonic acid

**DOI:** 10.1098/rsos.181363

**Published:** 2018-12-12

**Authors:** Lisha Zhong, Stewart F. Parker

**Affiliations:** 1 University of Cambridge, Downing College, Regent Street, Cambridge, Cambs, CB2 1DQ, UK; 2 ISIS Facility, STFC Rutherford Appleton Laboratory, Chilton, Didcot, Oxon, OX11 0QX, UK

**Keywords:** methanesulfonic acid, inelastic neutron scattering spectroscopy, infrared spectroscopy, Raman spectroscopy, density functional theory

## Abstract

In this work, we have used a combination of vibrational spectroscopy (infrared, Raman and inelastic neutron scattering) and periodic density functional theory to investigate the structure of methanesulfonic acid (MSA) in the liquid and solid states. The spectra clearly show that the hydrogen bonding is much stronger in the solid than the liquid state. The structure of MSA is not known; however, mineral acids typically adopt a chain structure in condensed phases. A periodic density functional theory (CASTEP) calculation based on the linear chain structure found in the closely related molecule trifluoromethanesulfonic acid gave good agreement between the observed and calculated spectra, particularly with regard to the methyl and sulfonate groups. The model accounts for the large widths of the asymmetric S-O stretch modes; however, the external mode region is not well described. Together, these observations suggest that the basic model of four molecules in the primitive unit cell, linked by hydrogen bonding into chains, is correct, but that MSA crystallizes in a different space group than that of trifluoromethanesulfonic acid.

## 1. Introduction

**Figure 1.**
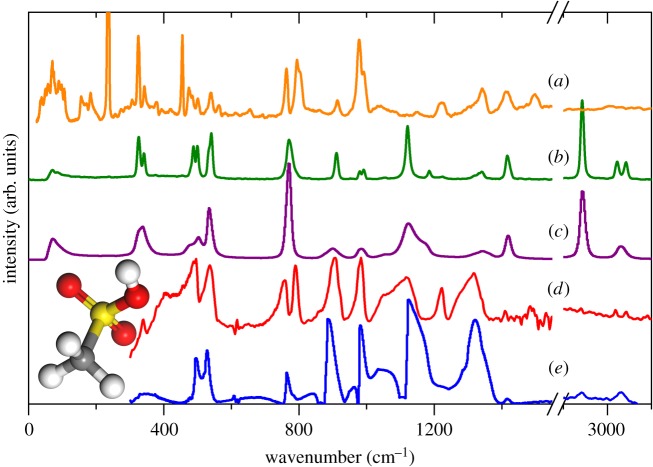
(*a*) INS, (*b*) FT-Raman (solid), (*c*) FT-Raman (liquid), (*d*) infrared (150 K, solid), (*e*) infrared (liquid, not corrected for ATR) spectra of methanesulfonic acid. The structure of methanesulfonic acid is shown in the inset (white, hydrogen; grey, carbon; yellow, sulfur; red, oxygen).

Methanesulfonic acid (MSA, see inset in figure 1 for the structure) is a molecule of interest particularly in atmospheric chemistry due to its formation by oxidation of dimethylsulfide (DMS, mainly produced by marine biota [1]) in the geochemical sulfur cycle. Although DMS accounts for less than 10% of the total non-sea-salt mass of aerosol sulfur, it is the only known source of MSA in marine air and could be a vital tracer for oceanic emissions and atmospheric reaction pathways of organic sulfur compounds [2]. This can then be used to determine the impact of sulfur-containing aerosols on the global climate [3]. In addition, it has found use in linking atmospheric conditions to natural events such as El Niño [1] as DMS emissions are significantly modulated by short-term as well as long-term climatic changes in the past, [4], particularly with regards to the ocean climate [5].

MSA has also been explored due to its environmental benefits (especially when compared to F- or B-containing alternatives) as it is readily biodegradable and can be considered to be a natural product, being part of the natural sulfur cycle. Moreover, it has good aqueous solubility, low toxicity and is easy to waste treat. Its high electrolyte conductivity requires less electricity and hence lower fuel consumption for electrochemical processes, in particular, as the commercial electrolyte standard for Sn/Pb solder electroplating (although it also has emerging uses in electroplating pure tin on sheet steel) [6].

As a catalyst, MSA has also been widely used in many pathways, for example, in cyclization reactions such as 3-arylpropanoic and 4-arylbutanoic acids to 1-indanones and 1-tetralones, respectively [7] and ring-opening polymerization, e.g. of trimethylene carbonate [8]. MSA is also a new and efficient catalyst for pathways such as the one-pot synthesis of 2-amino-4H-chromenes, [9] which have medical applications.

The vibrational spectroscopy of MSA has been studied for many years [10–14]. However, all of these relate to surface spectroscopy of aqueous MSA by vibrational sum frequency spectroscopy [10], or are of the gas or liquid states [11–13]: there are no published solid-state spectra. Here, we report a comprehensive study of the liquid and solid state of MSA, using a combination of infrared, FT-Raman and inelastic neutron scattering (INS) spectroscopies, with the assignments supported by density functional theory calculations.

## 2. Material and methods

### 2.1. Materials

MSA was used as received from Sigma-Aldrich (better than 99.0% purity).

### 2.2. Vibrational spectroscopy

The INS spectrum was recorded at less than 20 K using TOSCA [15] at ISIS (http://www.isis.stfc.ac.uk/). The spectrum is available at the INS database: http://wwwisis2.isis.rl.ac.uk/INSdatabase/. Infrared spectra were recorded using a Bruker Vertex70 FTIR spectrometer, over the range of 100–4000 cm^−1^ at 4 cm^−1^ resolution with a DLaTGS detector using 64 scans and the Bruker Diamond ATR. The use of the ultra-wide range beamsplitter enabled the entire spectral range to be recorded without the need to change beamsplitters. Variable temperature ATR infrared spectra (150–300 K) were recorded using a Specac Golden Gate accessory. The spectra have been corrected for the wavelength-dependent variation in path length using the Bruker software and also baseline corrected. FT-Raman spectra were recorded with a Bruker MultiRam spectrometer using 1064 nm excitation, 4 cm^−1^ resolution, 500 mW laser power and 64 scans. The liquid MSA Raman spectrum was measured in air at room temperature in a quartz cuvette; the solid MSA Raman spectrum was obtained by cooling the filled cuvette in liquid N_2_.

### 2.3. Computational studies

The plane wave pseudopotential-based program CASTEP was used for the calculation of the vibrational transition energies and their intensities [16,17]. The generalized gradient approximation (GGA) Perdew–Burke–Ernzerhof (PBE) functional was used in conjunction with optimized norm-conserving pseudopotentials. Electronic supplementary material, table S1 gives the details of the calculations. All of the calculations were converged to better than |0.0035| eV Å^−1^. After geometry optimization, the vibrational spectra were calculated in the harmonic approximation using density-functional perturbation theory [18]. This procedure generates the vibrational eigenvalues and eigenvectors, which allows visualization of the modes within Materials Studio (http://accelrys.com/products/collaborative-science/biovia-materials-studio/) and is also the information needed to calculate the INS spectrum using the program ACLIMAX [19]. We emphasize that the transition energies have *not* been scaled.

## 3. Results and discussion

Figure 1 shows a comparison of INS, infrared and Raman spectra obtained from the liquid and solid states. There are obvious differences between the solid and liquid states of MSA. In the Raman spectra, the peaks are sharpened and more readily resolved in the solid state, often changing from a single broader peak in the liquid state spectra to two distinct peaks in the solid state. The observed splitting of peaks can be explained by how the molecules become fixed in the solid, which destroys the symmetry seen in each individual molecule. Hence, the doubly degenerate peaks in the liquid state are no longer present in the solid state.

**Figure 2.**
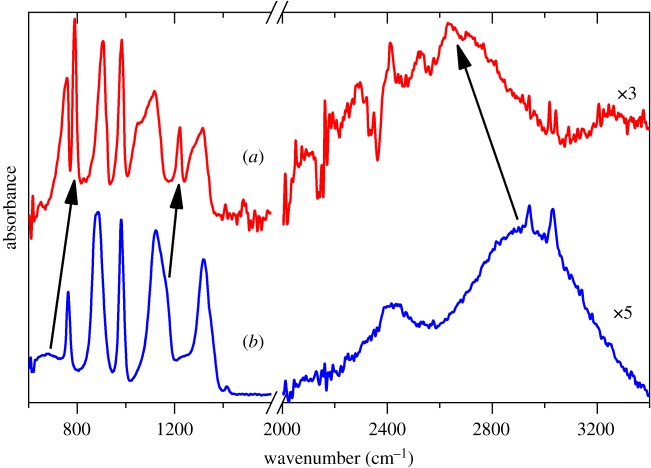
(*a*) Solid and (*b*) liquid infrared spectra of methanesulfonic acid. The arrows highlight the shifts due to the change in hydrogen bonding between the two states.

The effects of the hydrogen bonding are best seen in the infrared spectra, figure 2. As the hydrogen bonding strength increases, the O–H stretch peak downshifts and the S—O—H bending motions upshift. This can be seen in figure 2, where the broad O–H stretch peak in the liquid spectrum is downshifted from approximately 3000 cm^−1^ to 2600 cm^−1^ in the solid spectra. In addition, the 762 cm^−1^ (liquid) peak is upshifted to 790 cm^−1^ (solid) and the 1171 cm^−1^ shoulder (liquid) is resolved into the 1224 cm^−1^ peak (solid). Clearly, in the solid state, the hydrogen bond strength present in MSA increases.

The presence of hydrogen in MSA has a considerable effect on the spectra which can be seen by contrasting it with that of metal methanesulfonates [20]. Most of the modes are not affected and therefore appear at similar values. However, there is a large noticeable upshift of the SO_3_ symmetric and asymmetric stretching modes from approximately 1000 to 1150 cm^−1^ in the complexed methanesulfonate salts (Ag = 1018 and 1117, Cs = 1034 and 1177, Cd = 1058 and 1154, Cu = 1044 and 1140 + 1216) to 1088 and 1326 cm^−1^ in MSA.

Previous literature has suggested that MSA exists as a dimer structure with *C_i_* point group symmetry as a result of comparisons between calculations of frequency [12]. This conclusion may have been based on the well-known dimer formations present in carboxylic and benzoic acids [21]. However, there are exceptions to this in the form of monocarboxylic acids such as formic acid [22], acetic acid [23,24] and *β-*tetrolic acid [25], which preferentially form *catemer* motifs. This is possible as the molecules are small and non-bulky, therefore enabling them to pack within the limits of 5–8 Å that can be accommodated by the hydrogen bonds [26].

A linear chain structure is commonly found in mineral acids and, in particular, is seen in the closely related molecules fluorosulfuric acid and trifluoromethanesulfonic acid [27]. In the previous literature, nitric acid was also believed to exist as dimers in its solid state [28,29], but more recent findings are that it too follows a *catemer* motif [30], hence it is not unreasonable to question the dimer MSA structure given the advances in the understanding of these solid-state structures. Moreover, inconsistencies between the predicted spectra for a MSA dimer and the observations have even been noted in previous work [12].

**Figure 3.**
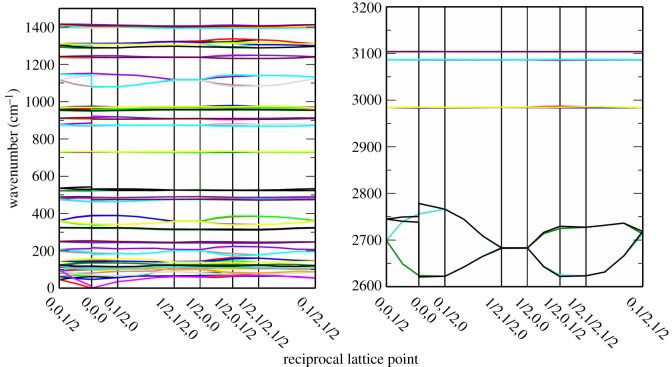
Calculated dispersion curves for the model structure of methanesulfonic acid.

**Figure 4.**
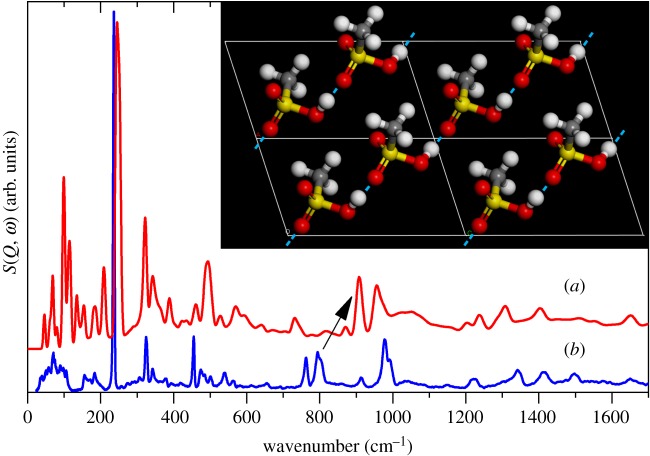
(*a*) Calculated and (*b*) experimental INS spectra of methanesulfonic acid. The arrow highlights the difference of the O—H out-of-plane bending mode due to the hydrogen bonding. The inset depicts the geometry- and lattice-optimized MSA structure showing the *catemer* motif. The dashed blue lines represent the hydrogen bonds between each monomer.

The crystal structure of MSA is not known and the reasons for this are not clear. MSA is a liquid at room temperature, thus the procedure for obtaining crystals suitable for X-ray diffraction is more complex, although there are many examples in the literature of such materials. However, the crystal structure of the closely related trifluoromethanesulfonic acid, which is also a liquid at room temperature and for which the solid-state structure is known [27], was used as the basis for our model. The structure is monoclinic, space group *P*2_1_/c, with four molecules in the primitive cell. By replacing the F with H in each molecule, we were able to construct an approximate structure for MSA in the linear chain structure. This structure was then geometry and lattice parameter optimized, noting that the space group was maintained. This resulted in a reduction of cell volume from 465.0 to 385.8 Å^3^. Vibrational analysis revealed all real modes present across the entire Brillouin zone, showing that the structure is dynamically stable, figure 3; the final structure is shown as the inset in figure 4. The calculated density of the solid is 1.65 gm cm^−3^, the density of the liquid is 1.48 gm cm^−3^, the stronger hydrogen bonding in the solid state would probably increase the density above that of the liquid, further towards the calculated value, suggesting that the basic model with four molecules in the primitive cell is correct.

**Figure 5.**
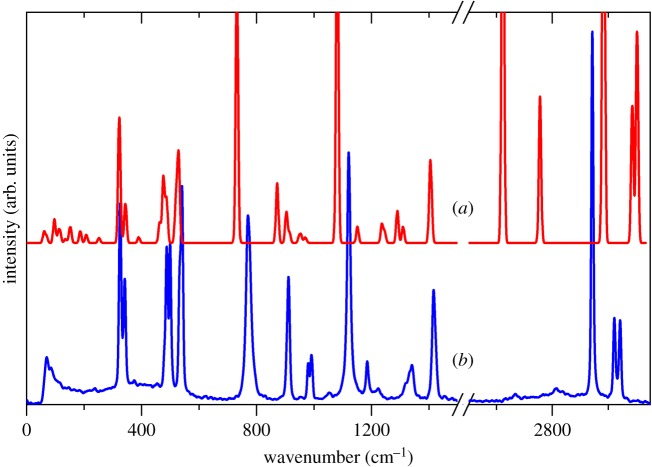
(*a*) Calculated and (*b*) experimental Raman spectra of methanesulfonic acid.

**Figure 6.**
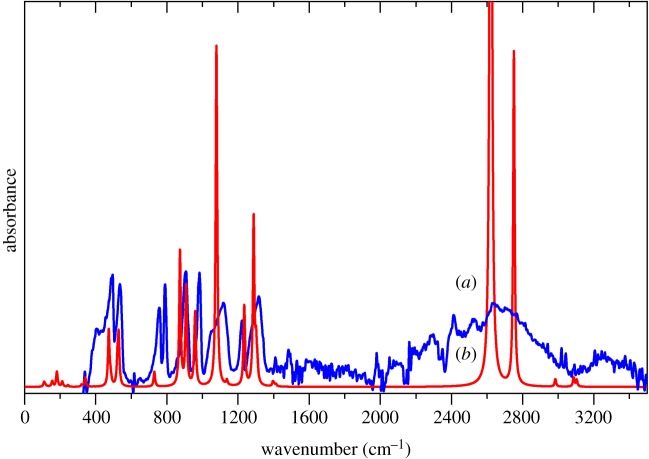
(*a*) Experimental and (*b*) calculated infrared spectra of methanesulfonic acid.

Figures 4–6 compare the observed and calculated INS, Raman and infrared spectra; table 1 lists the observed and calculated transition energies. From the INS comparison (figure 4), the main discrepancy in peak wavenumbers is due to hydrogen bonding. It is possible that this is a consequence of using CASTEP for the calculations, the reasons of which will be expanded upon later. The most striking feature of the INS spectrum is the massive CH_3_ torsion mode, which is well replicated in the calculations. Owing to this extremely high intensity, it is likely that strong overtones would be observed as additional peaks in the experimental spectrum. The fundamental and first overtone are easily seen, and the anharmonicity correction (harmonic frequency = *ω_e_*, anharmonicity = *x_e_ω_e_*) was calculated using the standard formulae [31] to deduce the locations of higher overtones. The values predicted (with *ω_e_* = 253 cm^−1^, *x_e_ω_e_* = 8.5 cm^−1^) were 236, 455 and 657 cm^−1^, which are clearly visible as the 236, 455 and 655 cm^−1^ peaks seen in the experimental spectrum, suggesting that the first, second and third overtones are present. The fourth overtone was predicted to appear at 842 cm^−1^ which is close to the weak feature lying at 860 cm^−1^. However, this is more likely to be a phonon wing from the S-O-H out of plane deformation. The small value of the anharmonicity correction, approximately 3% *ω_e,_* is consistent with weak intermolecular interactions. In methanesulfonate salts and complexes [20], the methyl group projects into empty space, as is also the case for trifluoromethanesulfonic acid [27] and this propagates through the calculation to our model structure. That this feature is conserved, suggests that it is also likely to occur in the real (unknown) structure.

**Table 1.** Transition energies (cm^−1^) of the internal modes of methanesulfonic acid. (v, very; s, strong; m, medium; w, weak; br, broad; sh, shoulder).

INS	Raman		infrared			
solid	solid	liquid	solid	liquid	calculated	description
	3043 m, 3024 m	3030 m	3039, 3022 vw	3028 vw	3104	CH_3_ asymmetric stretch
	2942 vs	2944 s	2939 vw	2942 vw	3086	CH_3_ symmetric stretch
	2815 vvw				2984	
	2666 vvw			2634 br	2688	O—H stretch
1499 m			1481 w		1409	
1412 m	1416 m	1417 m	1410 vw	1415 vw	1401	CH_3_ asymmetric bend
1340 m					1310	CH_3_ symmetric bend
	1340 w	1342 w	1326 m, br	1320 s	1297	SO_3_ asymmetric stretch
1225 m	1226 vvw	1177 sh	1223 m	1166 sh	1238	O—H bend in plane
	1120 s	1122 m	1088 m, vbr	1122 s	1112	SO_3_ symmetric stretch
975 vs, 992 s	990 w, 980 w	984 w	984 s	979 s	966	CH_3_ rock
					953	
913 w	911 m	898 w	906 s	875 sh, 886 s	909	S—OH stretch
794 s, 799 sh			790 s		872	O—H out-of-plane bend
762 s	770 s	769 vs	759 m	763 m	730	C—S symmetric stretch
655 vw						3×CH_3_ torsion
539 w, 563 vw	539 s	534 s	529 s	529 s	525	SO_3_ symmetric bend
475 w, 477, 479 sh, 499 w	488 s, 499 s	474 sh, 503	494 s	472 sh, 49 3s	485	SO_3_ asymmetric bend
455 vs						2×CH_3_ torsion
326 vs, 342 w	324 s, 342 m	325 sh, 339	339 s	329 w	365	SO_3_ rock
					323	
236 vv s					248	CH_3_ torsion
184 m						lattice modes
156 m						
71 s, br, 75, 77	70 w	72				

The Raman spectrum (figure 5) is consistent between both the observed and experimental data. This is because the features due to hydrogen bonding only appear weakly in the spectrum and the main features result from the CH_3_ and SO_3_ groups. However, there is a major miscalculation of intensity for the O–H stretch modes: in the calculation, these occur as extremely strong modes at 2622 and 2756 cm^−1^, whereas only a weak broad feature centred at 2821 cm^−1^ is seen in the experimental data. The very weak peaks seen at 2666 and 2815 cm^−1^ in the experimental spectrum are probably overtones of the symmetric and asymmetric methyl deformations enhanced in strength by Fermi resonance with the intense symmetric methyl stretch mode at 2942 cm^−1^.

The infrared spectrum (figure 6) appears to have a few inconsistencies between the calculated and experimental spectra, the most affected area appearing at 500–900 cm^−1^. It is important to note that the calculations did not take into account any line width mechanisms (e.g. electrical anharmonicity), which probably explains the difference in peak widths [32], especially for the SO_3_ symmetric and asymmetric stretching modes at 1088 and 1326 cm^−1^. Additionally, CASTEP does not incorporate hydrogen bonding well in its calculations, as found previously from work on metal methanesulfonates [20]. Therefore with this context in mind, the CH_3_ and SO_3_ modes match reasonably well. The broad peak due to OH has been calculated to be in the correct position, although this is likely to be fortuitous due to the cancellation of errors in the calculation [33].

**Figure 7.**
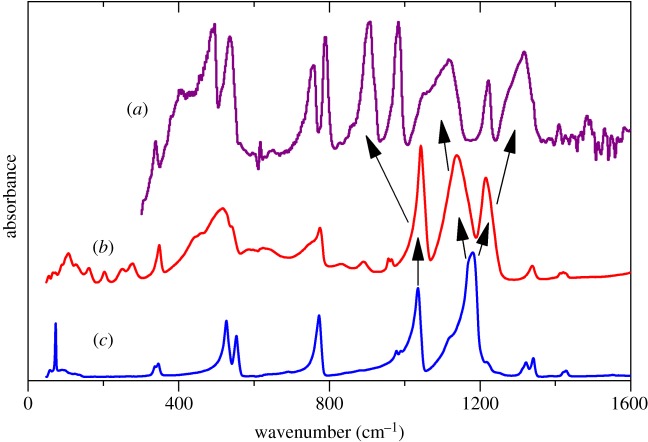
Infrared spectra of: (*a*) methanesulfonic acid, (*b*) copper methanesulfonate tetrahydrate and (*c*) caesium methanesulfonate. The arrows highlight the different peak locations of the S-O stretching modes due to intermolecular bonding involving the SO_3_ group.

We have previously investigated how the mode of coordination influences the vibrational spectra of methanesulfonates [20]. We found that only the asymmetric S—O stretch of the [CH_3_SO_3_]^−^ ion was systematically perturbed. Figure 7 compares the infrared spectra of MSA, Cu(H_2_O)_4_(CH_3_SO_3_)_2_ and Cs(CH_3_SO_3_). In the copper complex, the methanesulfonate is monodentate [34], and the caesium salt is ionic [35]. A proton can be considered as the extreme of coordination, and it can be seen that, in contrast to the previous examples, both the symmetric stretch and the asymmetric stretch modes are strongly affected: the splitting of the asymmetric stretch mode is more than twice as large as seen previously and the symmetric mode is downshifted by more than 100 cm^−1^. Visualization of the mode shows that while it is complex, also involving the C–S stretch and the methyl rock, only the S–OH bond is involved, the two nominally S=O bonds do not participate. This decoupling is not the result of the increased mass of the OH group relative to an O atom, as this would result in only a 4% or so shift. The large width of the components of the asymmetric mode are qualitatively reproduced by our model. Figure 3 shows the dispersion curves and it can be seen that at the Γ-point (0,0,0) the factor group splitting is 75 cm^−1^ for the mode at approximately 1100 cm^−1^, and 30 cm^−1^ for the mode at approximately 1300 cm^−1^, the experimental values are 106 and 62 cm^−1^.

## 4. Conclusion

We report the first spectra of MSA in the solid state. Comparison with the liquid state shows that the hydrogen bonding is stronger in the solid state, as shown by the upshift of the S-O-H deformation modes and the downshift of the O–H stretch mode. The solid-state structure of MSA is unknown; however, a periodic density functional theory (CASTEP) calculation based on the linear chain structure found in the closely related molecule trifluoromethanesulfonic acid gave good agreement between the observed and calculated spectra, particularly with regards to the methyl and sulfonate groups. The model accounts for the large widths of the asymmetric S-O stretch modes; however, the external mode region is not well described. Together, these observations suggest that the basic model of four molecules in the primitive unit cell, linked by hydrogen bonding into chains is correct, but that MSA crystallizes in a different space group than that of trifluoromethanesulfonic acid. The linear chain structure of MSA is probably retained in the liquid state, although with some disorder to account for the weaker hydrogen bonding.

## Data accessibility

The data which underpin this work are available via TopCat, the ISIS Facility's open access online data repository at: http://data.isis.stfc.ac.uk/doi/investigation/97991260.
